# Functional insights from targeted imaging BACE1: the first near-infrared fluorescent probe for Alzheimer’s disease diagnosis

**DOI:** 10.1186/s40824-022-00320-3

**Published:** 2022-12-09

**Authors:** Anyao Bi, Junyong Wu, Shuai Huang, Yongjiang Li, Fan Zheng, Jipeng Ding, Jie Dong, Daxiong Xiang, Wenbin Zeng

**Affiliations:** 1 Xiangya School of Pharmaceutical Sciences, Central South University, Changsha, 410013 PR China; 2 Department of Radiology the Second Xiangya Hospital, Central South University, Changsha, 410078 China; 3 Department of Pharmacy the Second Xiangya Hospital, Central South University, Changsha, 410078 China

**Keywords:** Alzheimer’s disease, Fluorescent probe, Near-infrared fluorescence imaging, BACE1, Materials imaging in vivo

## Abstract

**Background:**

β-Secretase (BACE1) is the vital enzyme in the pathogenic processes of Alzheimer's disease (AD). However, the development of a powerful tool with sensitivity for BACE1 determination in vivo is a challenge.

**Methods:**

A novel NIR fluorescent probe HBAE was synthetized from 2-hydroxy-3-methylbenzaldehyde and 2-amino-benzenethiol by 5 steps. The fluorescence mechanism in the ESIPT systems of HBAE probe was insighted with time-dependent density functional theory (TD-DFT) at the TDPBE0 level with the def2-TZVP approach. The corresponding docking between HBAE and BACE1 (PDB: 5I3Y) was performed through the ducking method by DOCK6.8. Then the BBB permeability of HBAE is verified by transwell orifice plate. 22-month-old male AD-model (5XFAD) mice and age-matched wild-type mice were employed to observe the brain kinetics by intravenous injection. Finally, Immunohistochemistry was performed on the AD brain section to reveal the levels of BACE1 in hippocampus and cortex areas and other regions in AD mice through the brain tissue slices by HBAE.

**Results:**

The NIR fluorescent probe HBAE was successfully applied in imaging BACE1 in AD model mice. The capability of HBAE in reflecting different level of BACE1 was performed by the specific imaging of the hippocampus region.

**Conclusions:**

We reported the first ESIPT near-infrared fluorescence probe HBAE for monitoring endogenous BACE1 in the AD live model mice, thus offering a versatile chemical tool for visualizing in the pathological processes of AD live brains. Remarkably, high resolution images showed the localization of red fluorescence stains in hippocampus of the AD brain. This study provides a promising way for functional insights from protein BACE1 in vivo.

**Supplementary Information:**

The online version contains supplementary material available at 10.1186/s40824-022-00320-3.

## Introduction

As one of the progressive neurodegenerative disorders in brain, Alzheimer’s disease (AD) has been regarded as an incurable condition [1–5]. Among various biomarkers, the formation and progressive accumulation of amyloid-β (Aβ) plaques in the brain is considered as an important pathological hallmark for diagnosis of AD at early stage [6–12]. Although thioflavin derivatives (ThT or ThS) are commercially available for in vitro histological staining amyloid fibrils [13–17], several inherent defects (e.g. distorted signals from fluorescence quenching effect at high concentration, inevitable noises from “always-on” mode, and poor blood-brain barrier (BBB) penetrability largely hinder their further application in in vivo imaging [18–20]. In fact, it is still far from accurate feedback for in situ visualization of Aβ plaques.

Generally, the Aβ peptide monomer is generated through the proteolysis of amyloid precursor protein (APP) by two typical proteases, β-secretases and γ-secretases [8–10]. Upon cleavage of APP by β-secretases, a soluble extracellular fragment (sAPPβ) is generated, and then its cell membrane-bound fragment (C99) undergoes a cleavage catalyzed by γ-secretase to form the Aβ monomer. As such, inhibiting the activity of β-secretases open a new window to limit the production rate of Aβ in vivo. In this way, β-secretase, also termed β-site APP-cleaving enzyme 1 (BACE1), shows a great significance in AD progression and provides a vital therapeutic target toward AD diagnosis and treatment.

To date, only a few fluorescent probes have been designed for detection of BACE1. For example, Franz et al. reported a probe based on fluorescence resonance the activities of energy transfer (FRET), allowing real time monitoring of the levels of BACE1 [21]. It was constructed through connecting DMACA with DABCYL quencher by a substrate of BACE which had a broad absorbance peak at 420–520 nm but faint fluorescence emission. Upon exposure to BACE1, by which the DABCYL quencher was removed, resulted in the fluorescence recovery of fluorophore DMACA. Tian’s group reported another two-photon fluorescent probe for BACE1 based on FRET as well [22]. The probe consists of an energy donor (mCyd) and an energy acceptor (AF633) conjugated by a peptide spacer which simultaneously behaves as a substrate of BACE1. Under the effect of BACE1, the signals of the probe changed from the fluorescence of AF633 to that of mCyd due to the separation of FRET donor–acceptor pair. Such a system is the first reported two-photon ratiometric fluorescent probe for imaging of BACE1 in living objects. However, NIR probe for BACE1 detection that can be permeable into blood-brain barrier are still quite rare nowadays.

Aggregation-induced emission (AIE) is a preferential method to design probes for the identification of protein fibrillogenesis, particularly account of its fluorescence emssion is so associated with the binding behavior during the aggregation process [23, 24]. Nevertheless, the redundant hydrophobic aromatic rings in such AIE probes and the additional π-conjugated bridge introduced to extend the emission wavelength to the NIR region, would undergo unwanted initial aggregation before binding toward BACE1, inevitably leading to a “false-positive” fluorescence signal. Hence, it is urgent to overcome the dilemma which is to balance the lipophilic requirement for facilitating longer emission wavelength with the docking behavior from water to protein detection of BACE1. In this paper, we increased the water solubility of the AIE probes which have NIR emision materials to obtain a favorable miscibility in aqueous media, thus affording the fluorescence imaging of BACE1 with high sensitivity and fidelity. We envisioned that the integration of deep penetration characteristic and tunable light-up fluorescence in such NIR AIE-active probes could gain unprecedented progress to directly visualize BACE1 deposition in vivo. Except for the difficulties mentioned above, photobleaching, tissue autofluorescence, and the detailed probes concentration will also cause a certain amount of background signals such as blood and tissue, which makes the fluorescent imaging working only on single short wavelength channel not reliable. While the excited state intramolecular proton transfer (ESIPT) is an important mechanism for constructing ratiometric fluorescent probes which might be a solution to the predicament. Besides, ESIPT has received considerable attention due to its large Stokes shifts, intensive absorption and emission in the UV/VIS region, and dual emission behaviour.

Under this circumstance, we developed a novel NIR fluorescent probe HBAE ((Z)-4-(4-(2-(3-(benzo[d]thiazol-2-yl)-4-hydroxy-5-methylphenyl)-1-cyanovinyl)phenyl)-1-methylpyridin-1-ium) based on the mechanism of AIE and ESIPT to image BACE1 in vitro and in vivo. A lipophilic π-conjugated benzene-bridge is conjugated to the ESIPT nuclear parent to extend the emission to NIR wavelength range. Then HBAE is obtained by the connection of pyridinium to the benzene-bridge. Consequently, the target of meeting the lipophilic requirement for extending the emission wavelength and avoiding a “background-positive” fluorescent signal in BACE1 imaging is realized with our probe. In vitro and in vivo experiments have provided evidences of the accurate imaging of intracellular BACE1. The imaging capacity of the probe towards BACE1 is firstly approved in cells. Then the BBB permeability of HBAE is verified before the in vivo assays. The feasibility of the probe is futher confirmed in the 22-month-old male AD-model mice. Finally, the result that the activities of BACE1 in hippocampus and cortex areas is higher than those in other regions in AD mice is intuitively shown through the brain tissue slices by HBAE. It is hoped that HBAE can serve as an efficient alternative to the NIR AD probes and provide a robust sensing materials platform to investigte the role of BACE1 in the pathogenesis of AD. Herein, we reported the first ESIPT near-infrared fluorescence probe for monitoring endogenous BACE1 in the AD live brains, and provided a versatile chemical tool for visualizing the pathological process of AD and its diagnosis.

## Results

### Molecular design and insighted fluorescence mechanism

It is due to the cellular localization of the active BACE enzyme that inhibitors fail in their attempts to inhibit cellular activity. When exposed to an extracellular environment, BACE is inactive at pH 7.4, so may not cause the cleavage of APP. A pH value of four to five is required for BACE1 to acquire activity once it is endocytosed to an endosome [11]. BACE1 cannot be inhibited by inhibitors that cannot access these endosomal compartments. Due to limited accessibility to intracellular vesicles, conventional FRET probes are ineffective as well for monitoring BACE1 activity in vitro.

Fluorophore’s molecular design and on achieving excitation coefficients and optimal excitation/emission spectra have attracted an increasing experimental interest, but few efforts are devoted to fundamental mechanistic studies. In this paper, we in-sighted the fluorescence mechanism in the ESIPT systems of HBAE probe with the ideal quantum chemical tools. Using time-dependent density functional theory (TD-DFT), we explore the potential energy surfaces (PESs) of the lowest-lying excited states rather than analysing frontier orbital energy diagrams and performing ESIPT thermodynamics calculations and the radiative and nonradiative decay rates from the involved excited states are computed from first-principles using a thermal vibration correlation function formalism [25–27]. With such strategies, our results reveal the real origins of the fluorescence intramolecular proton transfer.

**Fig. 1:**
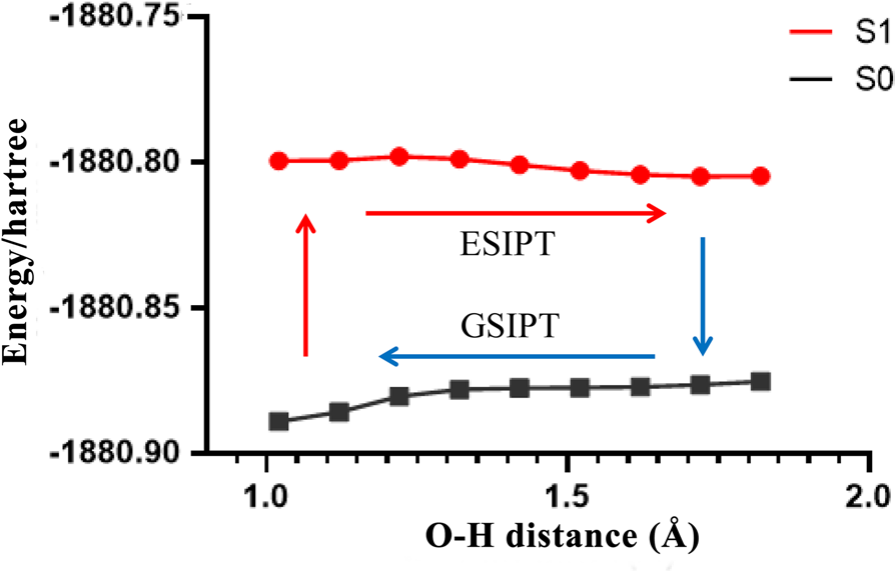
The calculations of mechanism of the HBAE fluorescence ESIPT processes, using the electronic-structure calculations in combination with the TDPBE0 level with the def2-TZVP approach

The emission mechanism of these fluorescence plays an important role in the molecular design and construction of a functional system. We simulated three possible nonradiative decay channels from the S1-state Franck−Condon structure of Z-enol, then consuming its excited-state energy and the ESIPT process along the O−H distances from Z-keto*. The PBE0/Def2-TZVP optimized S1-MEPs, are shown in Fig. 1. The computational results indicate that the probe being excited to the S1 state, a nonradiative decay from the excited to the ground state was observed and barrierless ESIPT from enol to keto tautomer in the waterable solution.

According to the reported structure, Our calculations confirm Z-enol as the most stable configuration in the S0 state at the PBE0 level. we calculated configurations of HBT the vertical absorption energy at Z-enol including Z-keto, E-enol, and E-keto to predict its absorption spectra. The S0 → S1 excitation leading to a spectroscopically bright state with π−π* character, mainly results from the HOMO to LUMO excitation. The S0 → S1 vertical excitation energy calculated is 540 nm (3.85 eV, f = 0.46) by TD-PBE0 with def2-TZVP approach. Both of those results are in agreement to 540 nm the experimentally measured absorption maximum and nano character (Figure S1-3 in supplementary information).

The tautomerized product, Z-keto*, shows vertical emission energy of 650 nm (2.53 eV) at the TDPBE0 level with the def2-TZVP approach (650 nm, 2.50 eV with SS-PCM calculation), which is in excellent agreement with the experimentally fluorescence maximum of 650 nm. Therefore, Z-keto* is assigned as the most likely emissive structure on the S1 state. In short, using the electronic-structure calculations in combination with the TDPBE0 level with the def2-TZVP approach, we have comprehensively investigated the possible emission channels of HBAE, thus confirming that fluorescence emission is owing to the effect of the ESIPT and subsequent bond-rotation relaxation, both of which are essential.

### Mechanism studies

Inspired by the prominent fluorescent performances of HBAE towards neuro cells (Figure S4 and S5 in supplementary information), we were then unveiled the underlying mechanism of the probe specificity and activity. According to previous reports, the effects of many brain-targeting compounds are attributed to the strong and selective inhibition of BACE1 [3, 5, 19]. Thus, we hypothesized that the reason of the strong affinity of HBAE on neuro cells probably was the strong targeting binding of it towards BACE1, by which the probe ultimately achieved specific target of neuro cells. In other words, as well demonstrated above that the probe could perform neuro cells specific bio-imaging, we conclused that the intracellular fluorescence intensity should be highly associated with the content of BACE1, and it in turn could signal the location of the targeting BACE1.

**Fig. 2:**
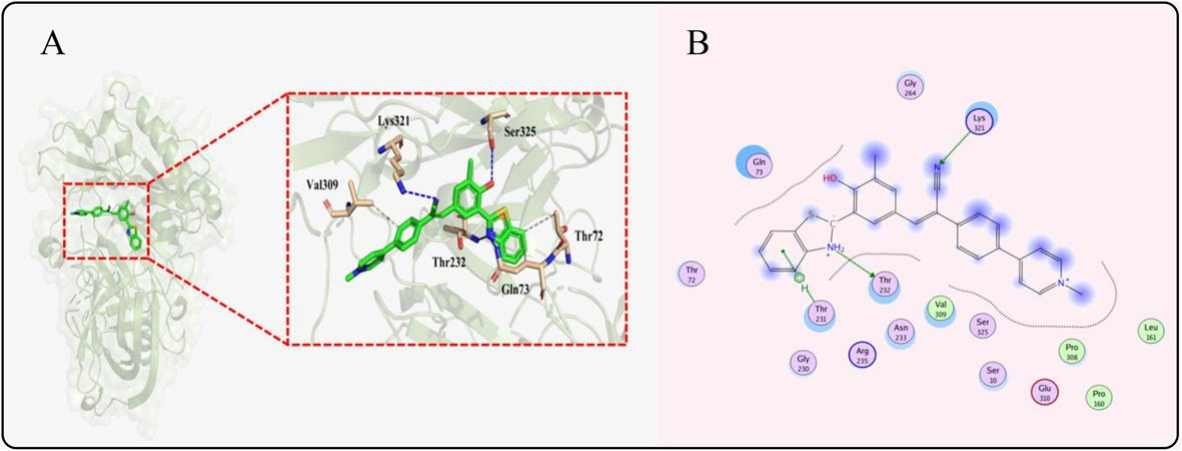
Illustration for the working principle of the designed NIR ratiometric fluorescent probe HBAE for the determination of BACE1 in neurons and mouse brain tissue slice. **A** the 3D docked conformation of the BACE1 and HBAE of the X-ray structure of BACE1. **B** the 2D docked conformation of the BACE1 and HBAE of the X-ray structure of BACE1 (PDB entry 5I3Y)

To prove our thoughts, the corresponding docking between HBAE and BACE1 (PDB: 5I3Y) was carried out initially. It was performed through the ducking method by swiss predict. As depicted in Fig. 2 A, the benzene ring on benzothiazole had Van der Waals force with Thr72 and Gln73, while the benzene-bridge had the same interaction with Val309. It was shown that HBAE was inserted into a nonpolar binding cavity of BACE1. It was further reflected that the fragments of the hydroxyl group and the cyano group of the probe were docked into the catalytic site of BACE1. These groups were interacted with Ser325 and Lys321 through hydrogen bonds, respectively. Besides, the N atom on benzothiazole also had a strong hydrogen bond with the amino acid residues of BACE1, including Gln73 and Thr 232 in Fig. 2B. These results suggested that the targeting ability of HBAE towards neuro cells could be based on the specific binding between the probe and BACE1.

### Specific imaging of BACE1 in human and mouse cells

Then, BACE1 in different human and mouse cell lines (HEK293 is a human embryonic kidney cell, U87-MG is a human glioma cell, N2a and bEnd. 3 are mouse derived cells) was analyzed using western blot images (Figure S7 in supplementary information). As shown in Figure S5., BACE1 contents obviously increased in N2a, HEK293, U87-MG and bEnd.3, which is the component part of the blood-brain barrier. These results demonstrated that the levels of BACE1 were highly expressed in the four cell lines. Binding affinity was another important factor for probes to imaging the BACE1. After the addition of HBAE to the BACE1, the aggregates binding was formed and the corresponding NIR fluorescence enhancement(~6fold) was immediately found at 665 nm, demonstrating that HBAE showed specific binding affinity with BACE1. So, the highly sensitive NIR fluorescence response of HBAE with BACE1 made it promising materials for mapping BACE1 in AD mouse brain.

**Fig. 3:**
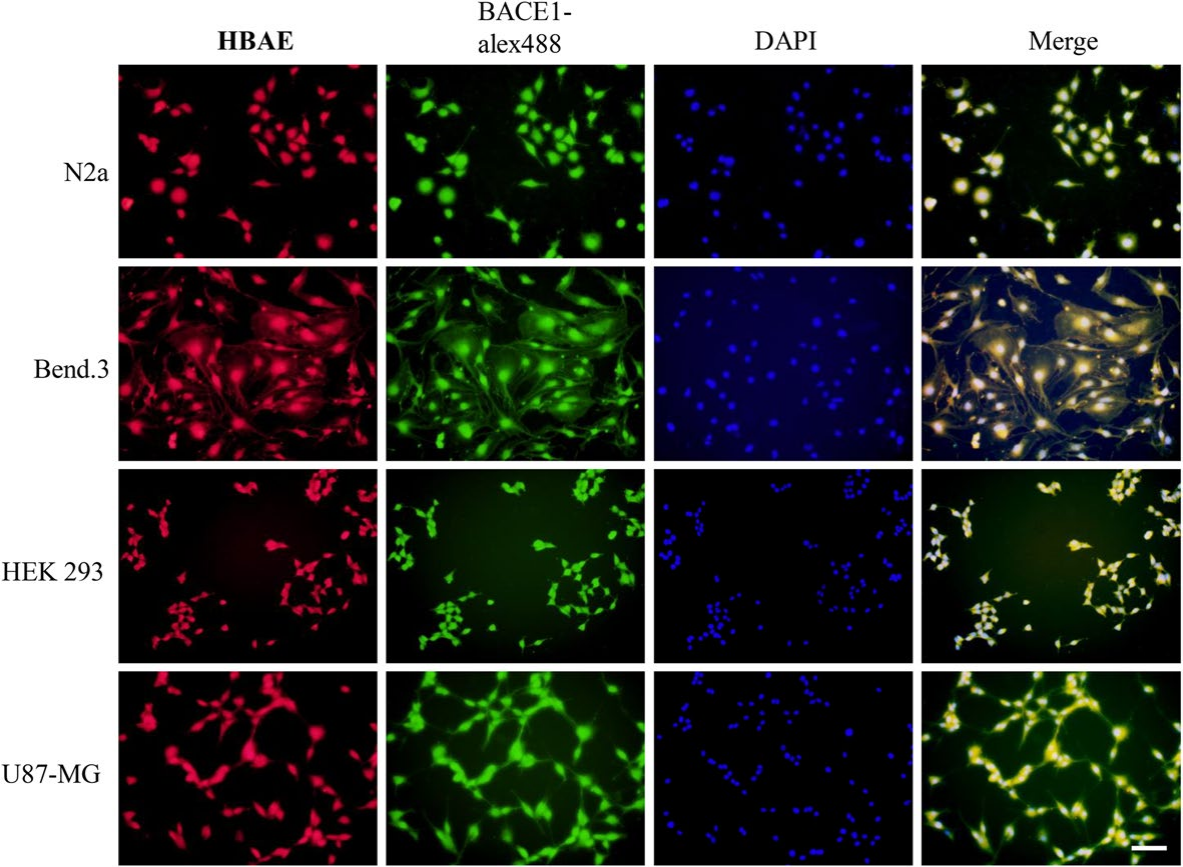
Imaging and sensing of endogenous BACE1 in four live cell lines by HBAE, Alex 488-BACE1 and DAPI. Scale bar = 50 μm

To detect and image BACE1 in the four cell lines, the four cells were stained with DAPI, HBAE and BACE1 specific antibody followed by the treatment with fluorescently (Alex fluo 488) labeled secondary antibody, and the images were captured under the fluorescence microscope (Fig. 3). Staining with Alex 488-BACE1, a specific antibody of BACE1, the green fluorescence is observed in the four cells. BACE1 is incubated with primary antibody binding and then decolored with green secondary anti-body (Alex fluo 488), so green fluorescence represents BACE1, which is highly expressed in four cell lines (Fig. 3). These results revealed that the four cell lines all have the high levels of BACE1, and HBAE can also be well imaged on cells and overlapped with BACE-1 specific antibody fluorescence. it provides a basis for the HBAE probe qualified endogenous BACE1 detection in human and mouse brain cells.

**Fig. 4:**
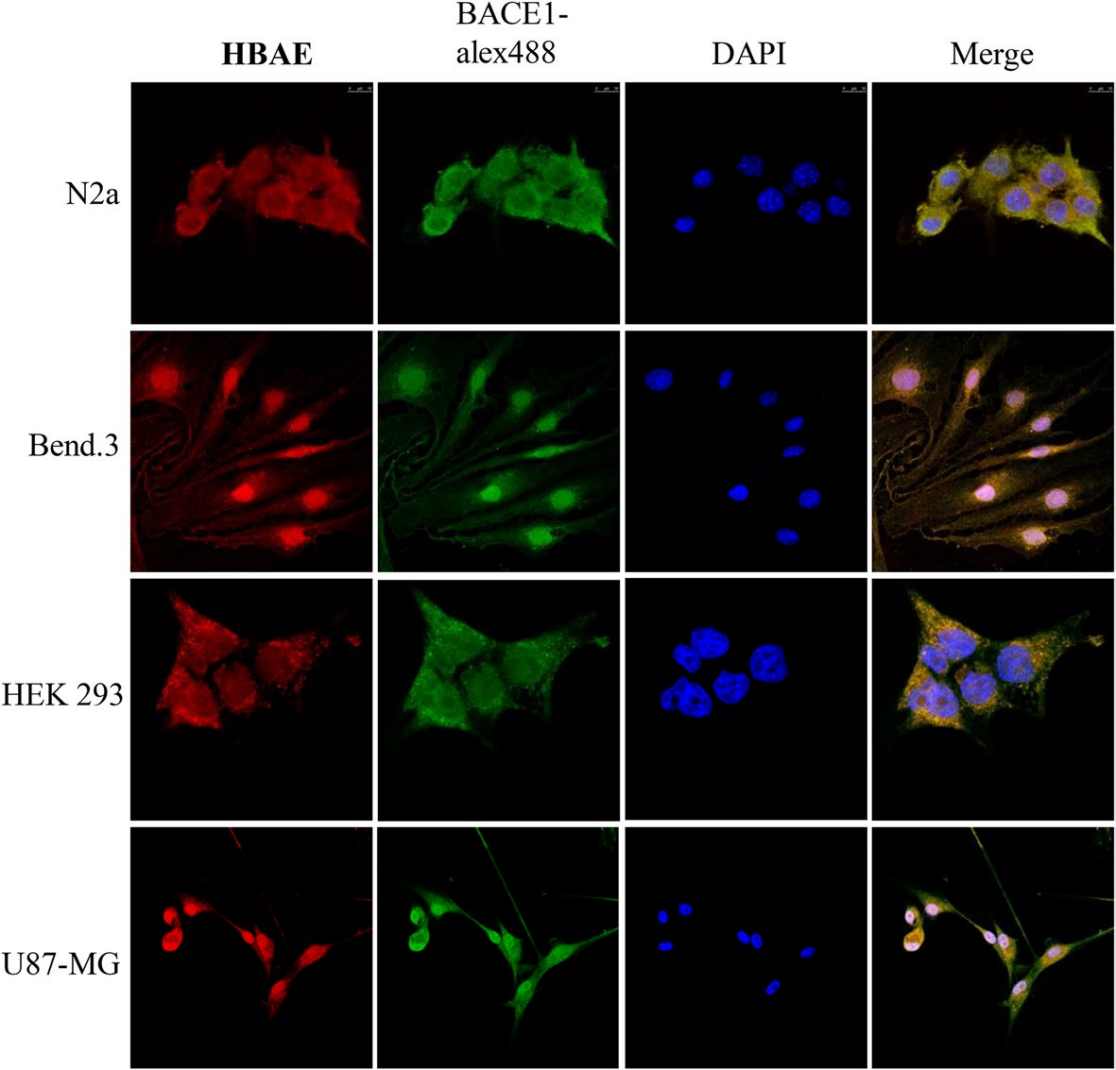
HBAE and BACE1 specific antibody (Alex fluo 488) followed by the treatment with fluorescently (Alex fluo 488) labeled secondary antibody. Scale bar = 10 μm

Next, for imaging and sensing of endogenous BACE1 in live human and mouse cells, 5.0 μM HBAE probe was incubated with cells for 20 min. From Fig. 4, we can see that the probe successfully entered into the human and mouse cells as shown in the overlay channel, the probe HBAE can be obvious observed from the merge image that it is well colocated with Alex fluo 488, indicating it can target BACE1 protein. The images obtained from the four cells showed green fluorescence signal which confirmed the presence of BACE1 mainly in the cell membrance and cytoplasm (Fig. 4), and demonstrating that the developed HBAE probe was quite qualified for endogenous BACE1 detection as expected.

### Evaluation of the blood brain barrier permeability

**Fig. 5:**
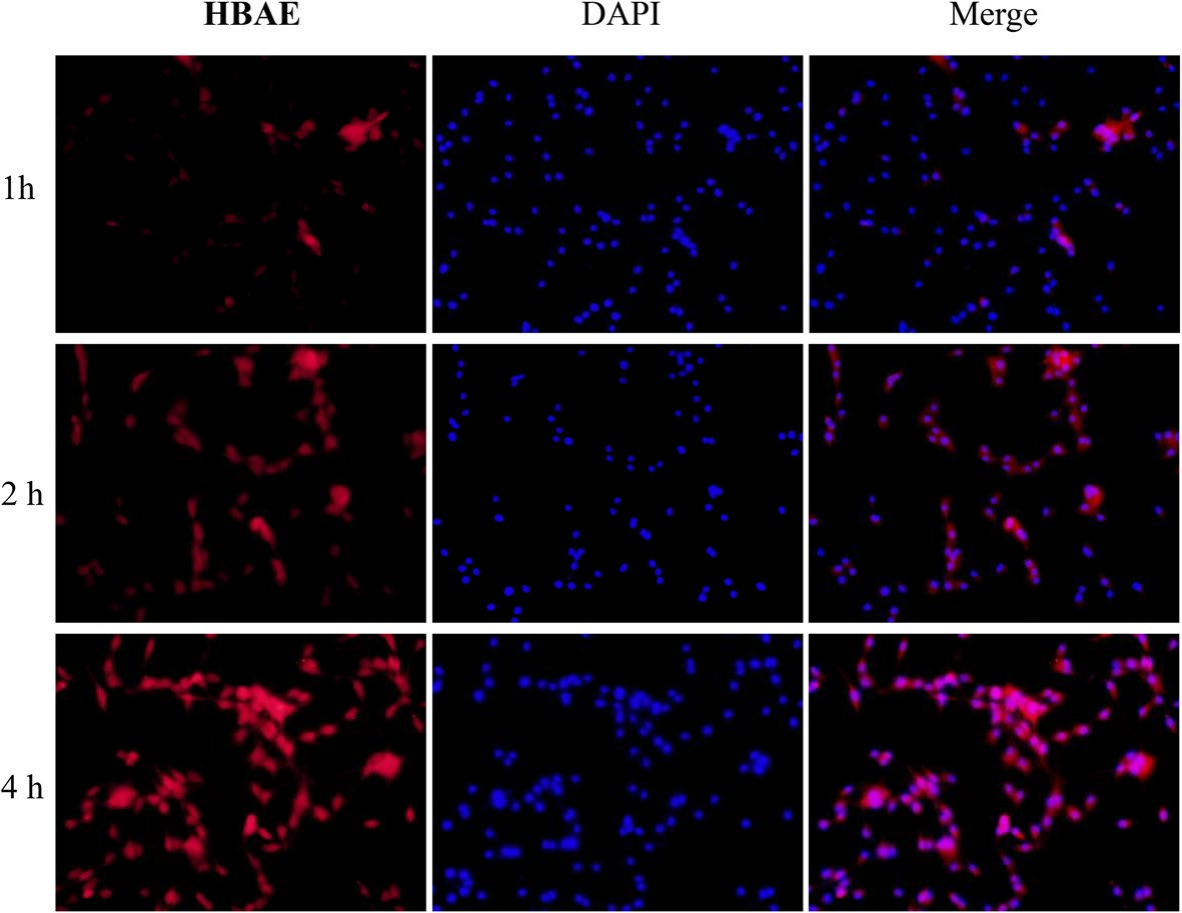
The penetrating blood-brain barrier ability of HBAE was evaluted by transwell experiment and showed that HBAE showed ideal permeability. U87-MG cells in the lower layer is obvious stained by HBAE. Scale bar = 50 μm

**Fig. 6:**
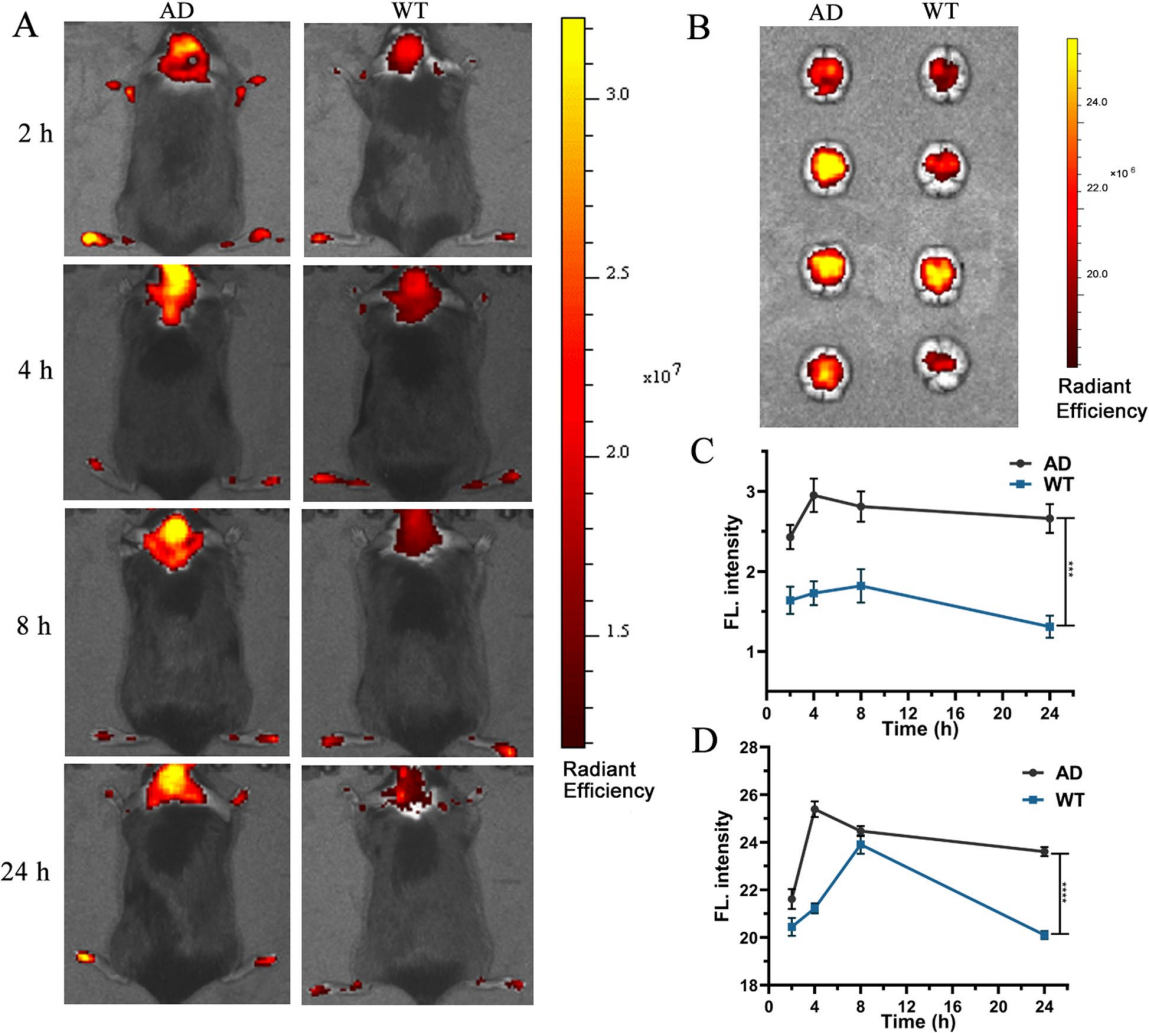
In vivo mapping of BACE1 in AD model (5XFAD) mice. **A** the fluorescent image at 2 h, 4 h, 8 h, 24 h after intravenous injection of 2.0 mg kg^–1^ of HBAE into wild-type mice and AD-model mice *in vivo*. **B** the fluorescent image of ex vivo brain of wild-type mice and AD-model mice at 2 h, 4 h, 8 h, 24 h after intravenous injection of 2.0 mg kg^–1^ of HBAE. **C** Fluorescence intensity of wild-type mice and AD-model mice in vivo at 2 h, 4 h, 8 h, 24 h after intravenous injection of 2.0 mg kg^–1^ of HBAE (^***^P < 0.001). **D** Fluorescence intensity of ex vivo brain of wild-type mice and AD-model mice (^****^P < 0.001)

Having confirmed BACE1 specific NIR light-up responses in BACE1 highly expressed cells. we further comfirmed of the probe is proved by simulating the blood-brain barrier in vitro with Transwell plate (Figure S8 in supplementary information). The bEnd. 3 cells planted in the upper chamber to simulate the blood-brain barrier in vitro, and then U87-MG cells are planted in the lower chamber. It can be found that within four hours, strong fluorescence could be captured in U87-MG cells indicating that HBAE can cross smoothly and has good permeability, the fluorescence staining diagram that the staining effect of U87-MG cells in the lower layer is obvious, indicating that the probe can penetrate the in vitro BBB model. The Transwell experiment analysis further confirmed that HBAE showed ideal permeability (Fig. 5). and realize the staining of lower cells, indicative of its potential for matching BBB penetrability. It provides ideal results for HBAE further imaging in living animals. In fact, via intravenous injection of HBAE (perfused with PBS), high-resolution imagine from the brain homogenate extraction of wild-type mice could be obtained to verify the BBB penetrability (Fig. 6).

### In vivo imaging in the AD model

As shown in Fig. 6A, 22-month-old male AD-model (5XFAD) mice (Figure S9 in supplementary information) and age-matched wild-type mice were employed to observe the brain imaging by intravenous injection, to further confirm the practicability of HBAE for in vivo imaging BACE1. The strong fluorescence signals were observed in the brain position. In particular, the fluorescence intensity of HBAE in the brain regions of the AD-model mice was much higher than that in the control of wild-type mice at 120 min after postinjection, there is strong fluorescence at 2 h, and the fluorescence will be stronger with time lapse, and there is always a strong fluorescence at 24 h. In contrast, in wild-type mice, the fluorescence became very weak after 24 h. indicative of specifically trapping BACE1 in vivo with probe HBAE. In addition, the cell viability of the probe HBAE by the CCK8 assays demonstrated its lower toxicity (Figure S6 in the Supporting Information). As shown in Fig. 6B, the fluorescent image of ex vivo brain of AD-model mice was obviously higher than that in the wild-type mice at corresponding time. As shown in Fig. 6C and D, it is obviously that HBAE could cross the blood–brain barrier and image BACE1 in vivo.

**Fig. 7:**
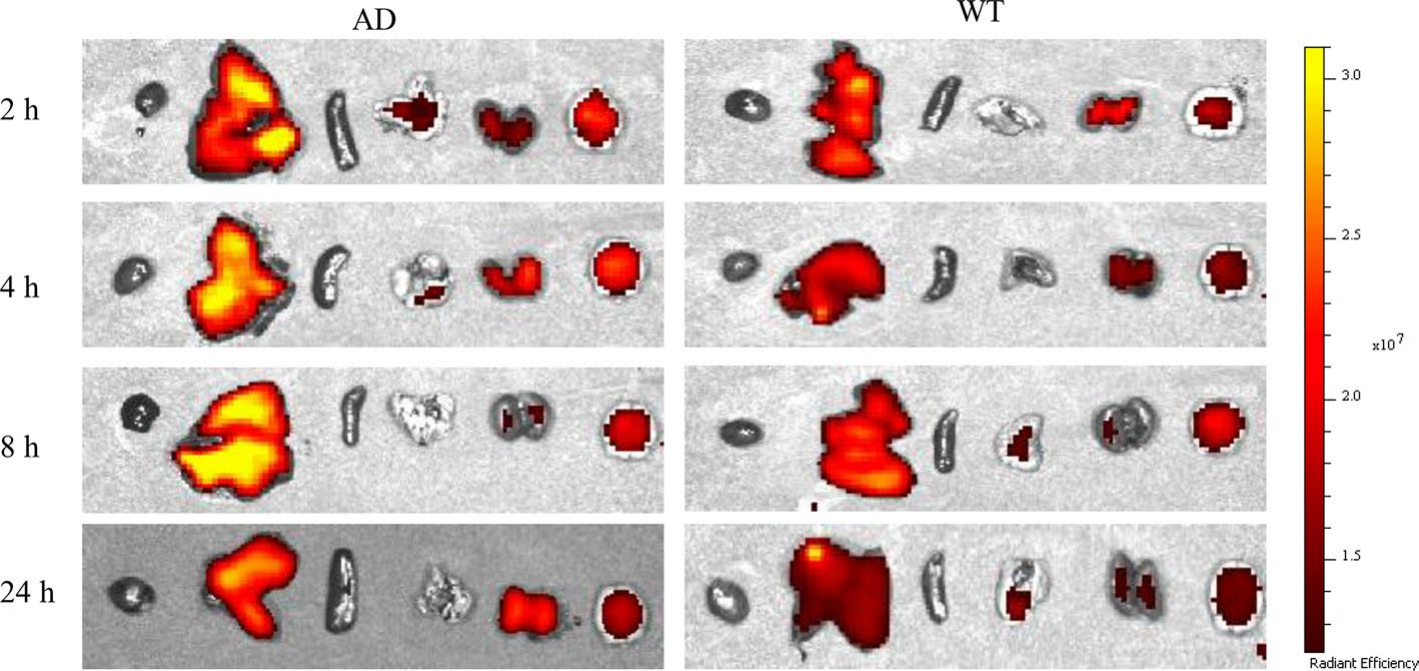
Ex vivo histology study of HBAE binding to BACE1 in AD and wild type mice. From left to right: heart, liver, spleen, lung, kidney and brain. According to the Figs. 6 and 7, most of compound HBAE has enter the brain, and probe is metabolized in the liver and cleared by the kidney

Ex vivo histology of HBAE binding to BACE1 in AD mice was carried out to further confirmed the in vivo performance. As shown in Fig. 7, after 120 min of intravenous injection of HBAE, a higher number of fluorescence was observed in the mouse brain,liver and kidney, indicating the probe mainly distribution in these organs. It was further confirmed that the in vivo fluorescence signal was resulting from HBAE specifically binding to BACE1.

### In vitro imagine of the hippocampus region in AD

**Fig. 8:**
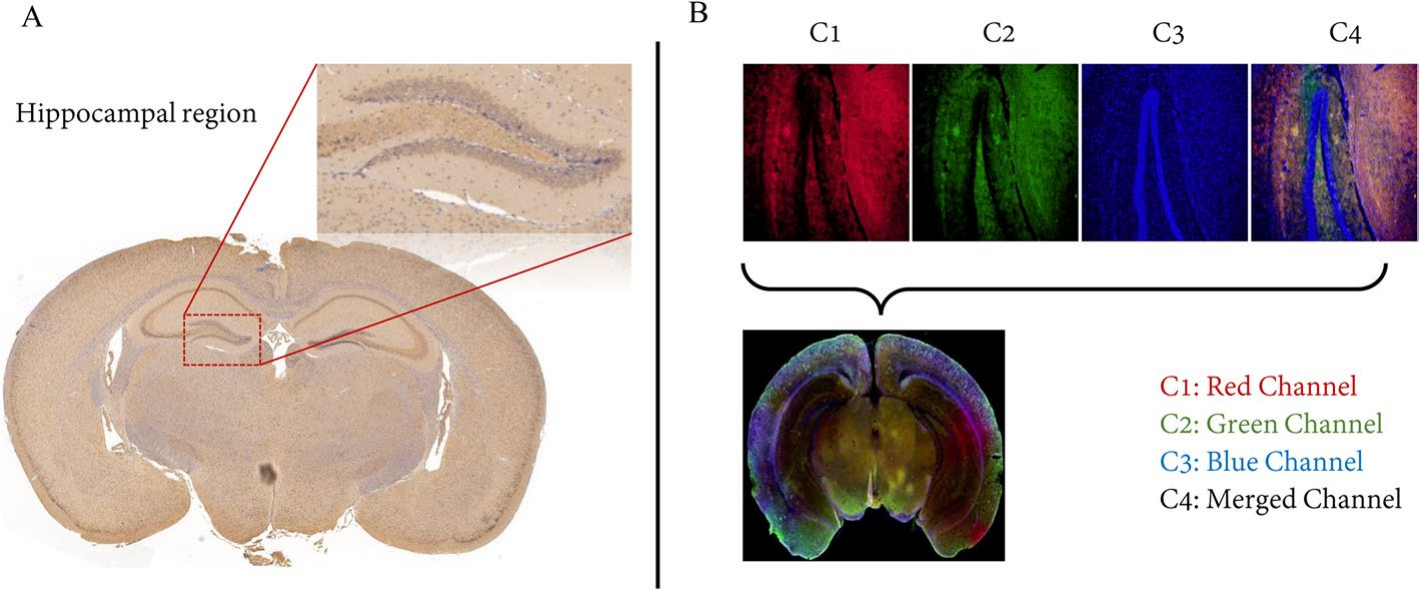
**A** the Immunohistochemistry with the BACE1-antibody in AD mouse brain, **B** the Immuno-fluorescence images of the hippocampus region in AD mouse brain tissue labelled with HBAE probe and Alex 488- BACE1. Illustration for multiple regions of mouse brain slices (C_1_: HBAE; C_2_: Alex 488- BACE1; C_3_: DAPI; C_4_: merged)

It is well-known that the areas of the brain such as the hippocampus and cortex, that are primarily involved in memory processing, are likely to be first affected by AD memory loss. Immunohistochemistry was performed on the AD brain section to see which part had high expression of BACE1, from the experimental results in Fig. 8A, it can be seen that the levels of BACE1 in hippocampus and cortex areas were higher than those in other regions in AD mice model. The images under red fluorescence channel revealed that the probe HBAE can well target proteins in AD mouse brain slices.

Next, the microscope images of the hippocampus region in AD mouse brain tissue labelled with the synthesized probe were obtained. After 120 min of intravenous injection of HBAE, the brain slice was obtained and incubated with BACE1 antibody, staining with secondary antibody, and finally stain the nucleus, a higher number of BACE1 were observed in the brain slices from 5XFAD mice (Fig. 8). As shown in Fig. 8B, the probe HBAE showed the excellent colocalization of BACE1 during staining the same section with BACE1 antibody (5.0 μM) was subsequently used for evaluating the levels of BACE1 in AD (5XFAD) mouse brain tissues. Figure 8B illustrates different regions of the mouse brain slice, such as field of hippocampus. From Fig. 8B, the pseudocolor of the Fgreen/Fred channel changed from green to red in the regions hippocampus and their arounds, demonstrating that BACE1 in these regions of AD mouse brain was higher than other regious. As shown in Fig. 8A, BACE1 obviously stained in hippocampus areas by BACE1-antibody, compared with those in other areas in AD mouse brain. As shown in Fig. 8B, HBAE could well staining the highly expressed BACE1 in the hippocampus area after entering the mouse. These results demonstrated that the levels of BACE1 were nonuniform in different regions of AD mouse brain. From the experimental results in Fig. 8, the mapping of fluorescent imaging of BACE1 in hippocampus and cortex areas was obviously beyond regions in AD mice, which can be regarded as that BACE1 levels were closely related to the pathogenesis of AD.

## Discussion

Alzheimer’s disease (AD) has been considered an incurable condition causing a progressive neurodegenerative brain disorder. Production of Aβ results from sequential cleavage of a transmembrane protein called amyloid precursor protein (APP) by β-Secretase (BACE1), which is the vital enzyme in the pathogenic processes of AD. However, the development of a powerful tool with high selectivity and sensitivity for BACE1 determination in vivo is a challenge in understanding the pathogenesis of AD. Therefore, the development of an effective fluorescent probe for tracking and monitoring BACE1 in biological systems is urgently needed.

In this study, we reported the first near-infrared (NIR) ESIPT fluorescence probe of its kind capable of monitoring dynamic changes of endogenous BACE1, through introducing a lipophilic π-conjugated thiophene-bridge for extension to NIR wavelength range with enhancement of BBB penetrability, thus making a breakthrough in high-fidelity feedback on in vivo detection of BACE1 with remarkable binding affinity in the AD live brains. We further insight the origins of the fluorescence mechanism in some of these systems with state-of-the art quantum chemical tools. Emission channels of HBAE were comprehensively insighted verified by the computational approaches combined with electronic-structure calculations. TD-DFT calculation has been systematically performed and clearly unraveled the lighting mechanism of fluorescent probe HBAE.

The novel near-infrared fluorescent probe was successfully applied for imaging and sensing of BACE1 in live cells, the images obtained from the four cells showed green fluorescence signal which confirmed the presence of BACE1 mainly in the cell membrane and cytoplasm, and demonstrating that the developed HBAE probe was quite qualified for endogenous BACE1 detection as expected. The first near-infrared fluorescent probe was deliberately constructed for AD-related BACE1 detection and imaging. The enhancement of BBB penetrability of HBAE was observed and provides ideal results for HBAE further imaging in living animals. The probe was successfully applied in monitoring endogenous BACE1 in vivo for the first time, indicating great potential to apply in the diagnosis of neurodegenerative disease. In order to offer potential solutions to further improve the clinic translation potential for the probes, we carried out the in vivo toxicity experiments for the novel probe HBAE as shown in Figure S10 in the Supporting Information. In order to study toxicity or biocompatibility results for cellular or in vivo use of developed HBAE probe, the hemolysis test of HBAE was also evaluated carefully. Meanwhile, In vivo safety evaluation and H&E staining of the AD brain have also been performed (Figure S11-S13 in the Supporting Information). Imaging of the hippocampus region was successfully performed to offer different level of BACE1 in brain regions, indicating BACE1 is an important biomarker for AD.

## Conclusion

In summary, we reported the first ESIPT near-infrared fluorescence probe of monitoring endogenous BACE1 in the AD live brains, thus offering a promising chemical tool for visualizing in the pathological processes of AD live brains. In vitro and in vivo experiments the probe provided ideal results of the accurate detection and mapping of BACE1. The first time NIR emission BACE1 was observed specificity, making a breakthrough in detection of BACE1 in vivo. The probe HBAE exhibited remarkable binding affinity with BBB penetrability, and high-performance NIR emission. The novel near-infrared probe was successfully applied for imaging and sensing of BACE1 in live cells, AD brain tissues and AD mice in vivo for the first time. Remarkably, high resolution images showed the localization of green fluorescence around red fluorescence stains in both hippocampus and cortex regions of the AD brain. This study provides an efficient alternative to the commercial probes serving a promising way for the imaging BACE1 by NIR probe and a new pathway for insights into protein BACE1 in vivo.

### Experimental section

#### Materials

All chemicals and reagents were used as received useless otherwise specified. Vitamin B1, benzaldehyde, and *p*-chloroaniline (99.5%) were purchased from Energy Chemical Co., Ltd (China). 4-Pyridine carboxaldehyde (98%) and pyridine were purchased from Sinopharm Chemical Reagent Co., Ltd (China). 4’,6-diamidino-2-phenylindole (DAPI) were purchased from Sigma-Aldrich. The high sugar DMEM base used was purchased from Thermo Fisher technology, phosphate buffered saline (PBS) were purchased from Invitrogen. The cell counting kit-8 (CCK-8) cytotoxicity assay kit was a commercial product of Beyotime Biotechnology (China). Milli-Q water was supplied by Milli-Q Plus System (Millipore Corporation, United States). The 22-month-old 5XFAD mice and wild-type mice were ordered from the Jackson Laboratory (34840) (Animal studies were performed according to the protocols approved by the Institutional Animal Care and Use Committees of the Department of Laboratory Animals of the Second Xiangya Hospital of Central South University), and maintained under standard conditions.

Cell lines: Murine derived Endothelial cells.3 (bEnd.3) and N2a, HEK293, U87-MG were maintained in Dulbecco’s modified Eagle’s minimum essential medium (DMEM) supplemented with 10% fetal bovine serum (Vivacell, Shanghai, China).

### Synthesis of HBAE

The probe HBAE was synthetized from 2-hydroxy-3-methylbenzaldehyde and 2-amino- benzenethiol via 5 steps (Scheme S1 in the Supporting Information). After Duff reaction and Suzuki reaction [28–30], the probe was given 0.32 g of yellow solid powder was obtained with a total yield of 19.2%. ^1^H NMR (500 MHz, DMSO-d6, δ): 9.05(s, 1H), 8.03-8.09(d, 2H), 7.70-7.71 (d, H), 7.63 (s, H), 7.59(d, H), 7.58-7.55 (d，2H), 7.51-7.45 (d, 2H), 7.41-7.39 (d, 2H), 7.32-7.30 (d, 2H), 7.22 (d, H), 7.18-7.14 (d, H), 4.03 (s, 3H), 1.34 (s, 3H). ^13^C NMR (125 MHz, DMSO-d6): δ): 168.35, 155.45, 153.58, 153.46, 151.47, 151.31, 146.17, 146.11, 144.10, 143.95, 137.96, 137.92, 134.02, 133.87, 133.18, 133.14, 129.31, 129.25, 127.67, 127.57, 127.04, 126.56, 124.46, 124.42, 123.01, 122.62, 118.50, 117.07, 47.40, 16.36. Mass spectrometry (ESI-MS, m/z): [M]^+^ Calcd. for [C_29_H_22_N_3_OS]^+^ 460.1515; found 460.1449. (Figure S14-S16 in the Supporting Information)

### Establishment of blood-brain barrier in vitro

Transwell orifice plate was used in the experiment. 10000 bend. 3 cells were inoculated into each well in the upper chamber. Complete culture medium was added into the lower chamber, and the solution was changed every 48 hours. The resistance was measured by trans endothelial resistance meter (TEER). When the resistance value was greater than 200 Ω / cm^2^, the in vitro blood-brain barrier was successfully established. U87-MG cells were inoculated into the lower chamber, adhered to the wall overnight, and HBAE were added into the upper chamber after 1, 2 and 4 hours respectively, The lower ventricular cells were fixed with paraformaldehyde, washed with PBS for three times, and stained with DAPI staining solution. After washing with PBS for three times, the BBB ability of the probe was observed under fluorescence microscope.

## Supplementary Information

**Additional file 1: Scheme S1.** Synthesis of Probe HBAE. **Figure S1.** UV-Vis spectrum and of fluorescent spectrum of HBAE in DMSO. **Figure S2.** Particle size of HBAE by dynamic light scattering. Average size=133.4 nm. **Figure S3.** TEM image of HBAE at pH=7.4. **Figure S4.** Fluorescence intensity of HBAE (10 μM) at 650 nm versus the different cells membranes protein. a. U87-MG cell membranes protein, b. N2a cell membranes protein, c. Bend.3 cell membranes protein, d.U251 cell membranes protein. The excitation wavelength was 560 nm. **Figure S5.** Fluorescence intensity of HBAE (10 μM) at 650 nm versus the different cells membranes protein. a. U87-MG cell membranes protein, b. N2a cell membranes protein, c. Bend.3 cell membranes protein, d. HEK293 cell membranes protein. **Figure S6.** CCK8 of the cell viability of HBAE. (Each sample was tested using three replicates, and the results are reported as the mean ± standard deviation). **Figure S7.** Western blot images of BACE1 in 4 cell lines. **Figure S8.** Supplementary information establishment of blood-brain barrier in vitro. **Figure S9.** Gene banding of AD and wild-type mice model. 1, 2, 3 and 6 belong to AD model mice, and 4, 5, 7 and 8 belong to wild-type mice. **Figure S10.** Test of hemolysis activity of formulations. (A) Photos of hemolysis after incubation with different formulations. HBAE of different concentrations are 12.5, 25, 50, 100, 200, 400 μ g/mL, respectively. **Figure S11.** H&E staining of heart, liver, spleen, lung, kidney tissues of C57 BL/6 mice after HBAE treatment, scale bar = 200 μm. **Figure S12.** Analysis of serum levels of ALT, AST, BUN and Cr in tumor-bearing mice after HBAE treatment. Data were mean ± SD (n = 5). **Figure S13.** The H&E staining of the AD mice brain after HBAE treatment. **Figure S14.** ^1^H NMR (500 MHz, DMSO-d6) of HBAE. Figure S15. ^13^C NMR (125 MHz, DMSO-d6) of HBAE. Figure S16. MS spectra of HBAE.

## Data Availability

The datasets used and/or analyzed in this study are available from the corresponding author on reasonable request.
