# Supplementary material for: Functional insights from targeted imaging BACE1: the first near-infrared fluorescent probe for Alzheimer’s disease diagnosis
Source: Biomater Res. 2022 Dec 9;26:76. doi: 10.1186/s40824-022-00320-3 (PMC9733252; doi:10.1186/s40824-022-00320-3)
Supplement: Supplementary file 1 — Additional file 1: Scheme S1. Synthesis of Probe HBAE. Figure S1. UV-Vis spectrum and of fluorescent spectrum of HBAE in DMSO. Figure S2. Particle size of HBAE by dynamic light scattering. Average size=133.4 nm. Figure S3. TEM image of HBAE at pH=7.4. Figure S4. Fluorescence intensity of HBAE (10 μM) at 650 nm versus the different cells membranes protein. a. U87-MG cell membranes protein, b. N2a cell membranes protein, c. Bend.3 cell membranes protein, d.U251 cell membranes protein. The excitation wavelength was 560 nm. Figure S5. Fluorescence intensity of HBAE (10 μM) at 650 nm versus the different cells membranes protein. a. U87-MG cell membranes protein, b. N2a cell membranes protein, c. Bend.3 cell membranes protein, d. HEK293 cell membranes protein. Figure S6. CCK8 of the cell viability of HBAE. (Each sample was tested using three replicates, and the results are reported as the mean ± standard deviation). Figure S7. Western blot images of BACE1 in 4 cell lines. Figure S8. Supplementary information establishment of blood-brain barrier in vitro. Figure S9. Gene banding of AD and wild-type mice model. 1, 2, 3 and 6 belong to AD model mice, and 4, 5, 7 and 8 belong to wild-type mice. Figure S10. Test of hemolysis activity of formulations. (A) Photos of hemolysis after incubation with different formulations. HBAE of different concentrations are 12.5, 25, 50, 100, 200, 400 μ g/mL, respectively. Figure S11. H&E staining of heart, liver, spleen, lung, kidney tissues of C57 BL/6 mice after HBAE treatment, scale bar = 200 μm. Figure S12. Analysis of serum levels of ALT, AST, BUN and Cr in tumor-bearing mice after HBAE treatment. Data were mean ± SD (n = 5). Figure S13. The H&E staining of the AD mice brain after HBAE treatment. Figure S14. 1H NMR (500 MHz, DMSO-d6) of HBAE. Figure S15. 13C NMR (125 MHz, DMSO-d6) of HBAE. Figure S16. MS spectra of HBAE. [file 40824_2022_320_MOESM1_ESM.docx]

**Supporting Information**

Functional insights from targeted imaging BACE1: The first near-infrared fluorescent probe for Alzheimer’s disease diagnosis

Anyao Bi, ^#[a,c]^ Junyong Wu, ^#[b]^ Shuai Huang, ^[a]^ Yongjiang Li, ^[b]^ Fan Zheng, ^[a]^ Jipeng Ding, ^[a]^ Jie Dong^[a]^, Daxiong Xiang*^[b]^ and Wenbin Zeng*^[a]^

[a] Xiangya School of Pharmaceutical Sciences, Central South University, Changsha, 410013, PR China.

[b] Department of Pharmacy the second Xiangya Hospital, Central South University, Changsha 410078, China.

[c] Department of Radiology the second Xiangya Hospital, Central South University, Changsha 410078, China.

[#] These authors contributed equally to this work.

**Contents**

S1 Synthesis and characterization

S1.1 Materials and methods

S1.2 Fluorescence measurement

S1.3 The imaging of cells by **HBAE**

S2 Cell experiments

S2.1 Cell culture

S2.2 Cell Viability Assay

S2.3 Western blot analysis

S3 Animal experiment

S3.1 Animal Model

S3.2 Real-time in Vivo imaging in 5XFAD Mice of **HBAE**

**S1 Synthesis and characterization**

**S1.1 Materials and methods**

All chemicals and reagents were used as received useless otherwise specified. Vitamin B1, benzaldehyde, and *p*-chloroaniline (99.5%) were purchased from Energy Chemical Co., Ltd (China). 4-Pyridine carboxaldehyde (98%) and pyridine were purchased from Sinopharm Chemical Reagent Co., Ltd (China). 4’,6-diamidino-2-phenylindole (DAPI) were purchased from Sigma-Aldrich. The high sugar DMEM base used was purchased from Thermo Fisher technology, phosphate buffered saline (PBS) were purchased from Invitrogen. The cell counting kit-8 (CCK-8) cytotoxicity assay kit was a commercial product of Beyotime Biotechnology (China). Milli-Q water was supplied by Milli-Q Plus System (Millipore Corporation, United States).

^1^H and ^13^C NMR spectra were measured on a Bruker ARX 400 MHz NMR spectrometer using CDCl_3_ and DMSO-d6 as the solvent and tetramethylsilane (TMS; δ = 0) as the internal reference. UV-vis Absorption spectra were performed on a UV-2450 scanning spectrophotometer (Shimadzu, Japan). Fluorescent spectra were recorded on a Hitachi F-2700 equipped with a 1 cm quartz cell. Fluorescent images were collected on Leica DMI4000B fluorescence microscope. The AD mice were scanned by Bruker SkyScan 1178.

**Cell lines:** derived Endothelial cells.3 (bEnd.3) and N2a, HEK293, U87-MG were continuously cultured in our laboratory.


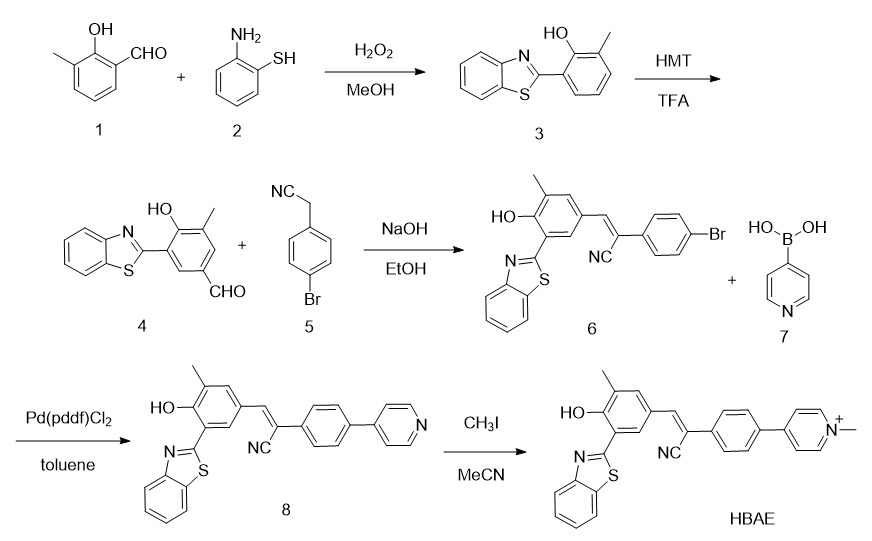


Scheme S1. Synthesis of the probe **HBAE**.

Synthesis of compound **3**

Compound **1** (1.96 g, 10 mmol) in a 250 mL round bottom flask was added with 40 mL methanol. The mixture was added with compound **2** (1.25 g, 10 mmol) and stir at room temperature for 5 hours. Then the reaction solution gradually changes from gray liquid to light yellow liquid, which was monitored by TLC. After 6 hours, the reaction solution was poured into 200 mL of water, precipitated a large amount of light-yellow solid. Filtering the reaction under reduced pressure and obtained light yellow solid, washing with saturated sodium chloride aqueous solution for 3 times, dry at 45 ^o^C in an oven. Then it dissolved in dichloromethane and recrystallized with cyclohexane to obtain 2.35 g of light-yellow solid powder, with a yield of 90.0%. ^1^H NMR (400 MHz, CDCl_3_, δ): 12.77 (s, 1H), 7.95 (d, 1H), 7.87 (d, 1H), 7.53 (d, 1H), 7.48 (d, 1H), 7.38 (d, 1H), 7.26–7.22 (m, 1H), 6.85 (t, 1H), 2.35 (s, 3H).

Synthesis of compound **4**

Compound **3** (0.241 g, 1 mmol) was dissolved in a round bottom flask and added with 3 mL of TFA and HMT (0.500 g, 3 mmol). The mixture was protected by in nitrogen, heated at reflux. After 4 hours, the reaction solution was dried and further purified by column chromatography to give a light-yellow powder (compound **4**, 0.212 g, yield 67.8%). ^1^H NMR (400 MHz, CDCl_3_, δ): 13.42 (s, 1H), 9.78 (s, 1H), 7.90–7.81 (m,3H), 7.63 (s, 1H), 7.48–7.34 (m, 2H), 2.32 (s, 3H).

Synthesis of compound **6**

Compound **4** (0.271 g, 1 mmol) and compound **5** (0.196 g, 1 mmol) were added into a round bottom flask, adding 5 ml of ethanol, then dropping 2 ml of a saturated aqueous solution of NaOH, stirring at room temperature. After 12 h, adding HCl to adjust the reaction solution into neutral, and further purified by column chromatography with PE/EA (20/1, v/v) giving 0.395 g of light yellow solid powder with a yield of 84.7%.^1^H NMR(500 MHz, DMSO-d6, δ): 9.05(s, 1H), 7.70-7.71 (d, H), 7.63 (d, 2H), 7.59(d, H), 7.51-7.45 (d, 2H), 7.41-7.39 (d, 2H), 7.35 (d, 2H), 7.22 (d, H), 1.34 (s, 3H).

Synthesis of compound **8**

Compound **6** (0.450 g, 1 mmol) and compound **7** (0.123 g, 1 mmol) were added into a round bottom flask, adding PdCl_2_(dppf) (10 mg, 0.02 mmol) under nitrogen protection and anhydrous conditions, then reflux for 12 hours at 90 ^o^C. After cooling, the precipitate was filtered, and further purified by column chromatography with PE/EA (3/1, v/v) to afford 0.245 g of yellow solid powder with a yield of 55.1%.^1^H NMR(500 MHz, DMSO-d6, δ): 9.05(s, 1H), 7.83-7.89(d, 2H), 7.70-7.71 (d, H), 7.63 (s, H), 7.59(d, H), 7.58-7.55 (d，2H), 7.51-7.45 (d, 2H), 7.41-7.39 (d, 2H), 7.32-7.30 (d, 2H), 7.22 (d, H), 7.18-7.14 (d, H), 1.34 (s, 3H).

Synthesis of (Z)-4-(4-(2-(3-(benzo[d]thiazol-2-yl)-4-hydroxy-5-methylphenyl)-1 -cyanovinyl)phenyl)-1-methylpyridin-1-ium (Probe **HBAE**)

Compound **8** (0.445 g, 1 mmol) was added to solution of acetonitrile (20 ml), pouring into a 100 ml round bottom flask. Dropping methyl iodide (0.110 g, 10 mmol) under the condition of nitrogen protection, and stirring at 90^o^C. After 12 hours, solution was dried and dissolved in dichloromethane, and purified by column chromatography with DCM/MeOH (10/1, v/v), and 0.32 g of yellow solid powder was obtained with a yield of 69.6%.^1^H NMR(500 MHz, DMSO-d6, δ): 9.05(s, 1H), 8.03-8.09(d, 2H), 7.70-7.71 (d, H), 7.63 (s, H), 7.59(d, H), 7.58-7.55 (d，2H), 7.51-7.45 (d, 2H), 7.41-7.39 (d, 2H), 7.32-7.30 (d, 2H), 7.22 (d, H), 7.18-7.14 (d, H), 4.03 (s, 3H), 1.34 (s, 3H). ^13^C NMR (125 MHz, DMSO-d6, δ): 168.35, 155.45, 153.58, 153.46, 151.47, 151.31, 146.17, 146.11, 144.10, 143.95, 137.96, 137.92, 134.02, 133.87, 133.18, 133.14, 129.31, 129.25, 127.67, 127.57, 127.04, 126.56, 124.46, 124.42, 123.01, 122.62, 118.50, 117.07, 47.40, 16.36. Mass spectrometry (ESI-MS, m/z): [M]+ Calcd. for [C_29_H_22_N_3_OS]^+^460.1515; found 460.1455.

**S3. Calculate the fluorescence quantum yield.**

Fluorescence quantum yield (Φf) was determined by using quinine sulfate (Φf = 0.58, in 0.1 M H_2_SO_4_ aqueous solution) as the fluorescencestandard.^1^ The quantum yield was calculated using the following equation. ΦF(X) = ΦF(S) (ASFX/AXFS) (nX/nS)^2^ Where ΦF is the fluorescence quantum yield, A is the absorbance at the excitation wavelength, F is the area under the corrected emission curve, and n is the refractive index of the solvent used. Subscripts S and X refer to the standard and to the unknown, respectively. The quantum yield of **HBAE** was calculated 0.27, respectively.

**S4.Absorption and Fluorescence emission spectra of HBAE**


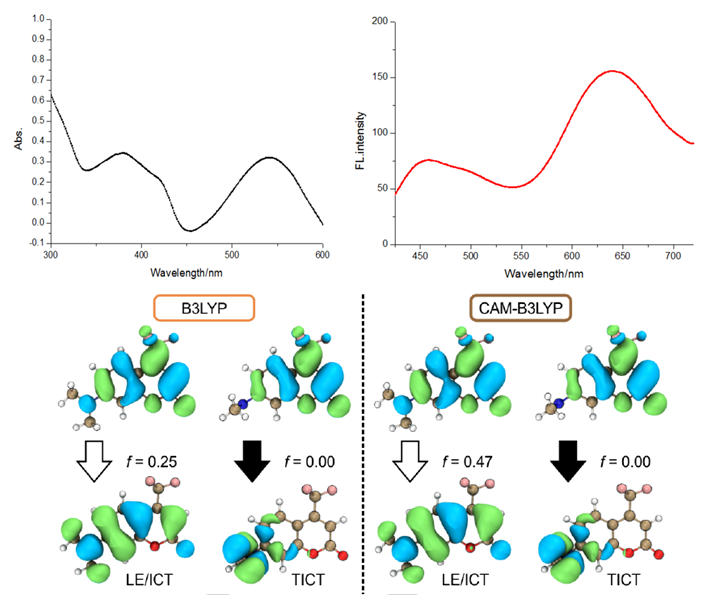


Figure S1. UV-Vis spectrum and of fluorescent spectrum of **HBAE** in DMSO.


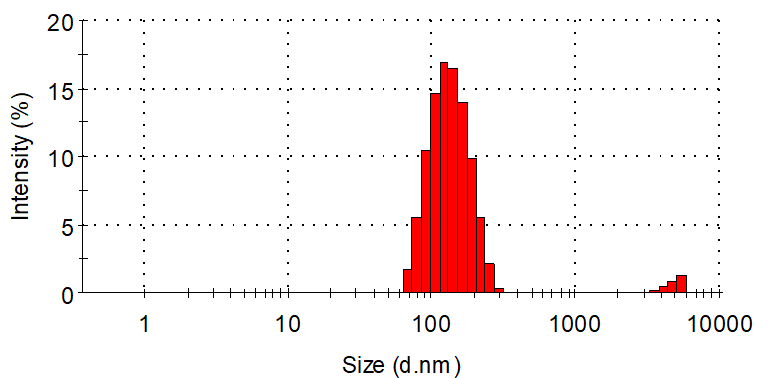


Figure S2. Particle size of **HBAE** by dynamic light scattering.

Average size=133.4 nm.


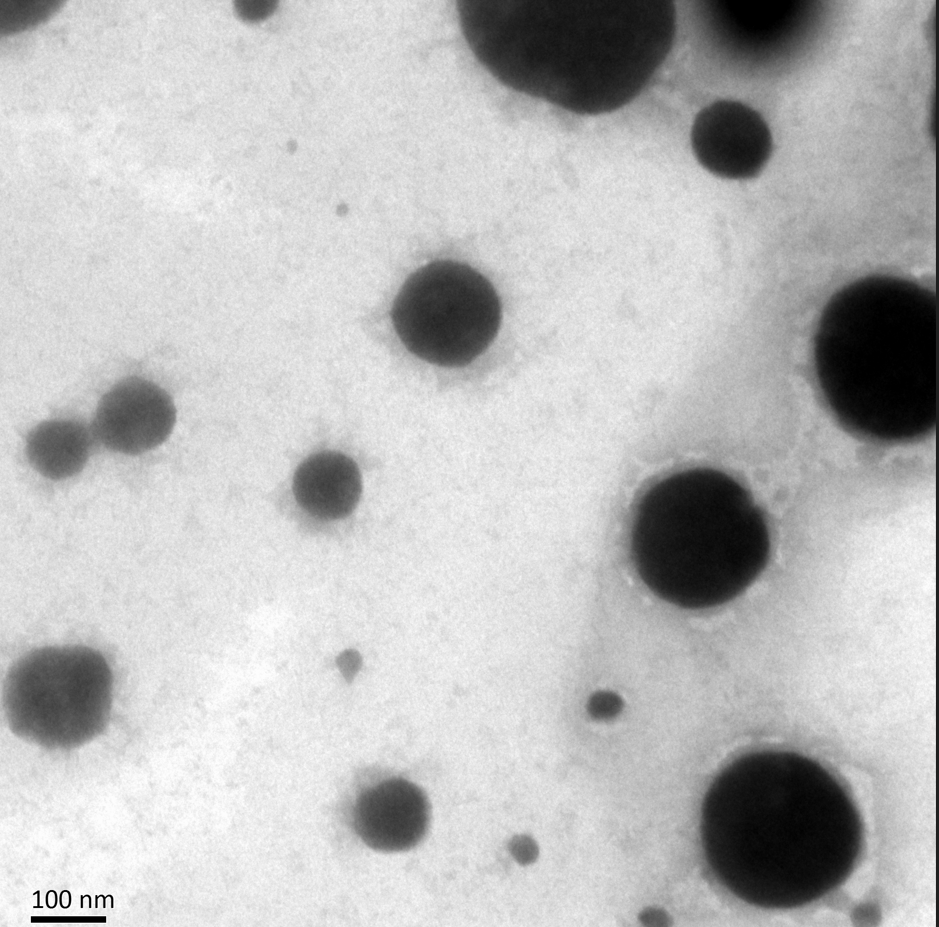


Figure S3 TEM image of **HBAE** at pH=7.4.

**S5. The imaging of cells membranes protein by HBAE**


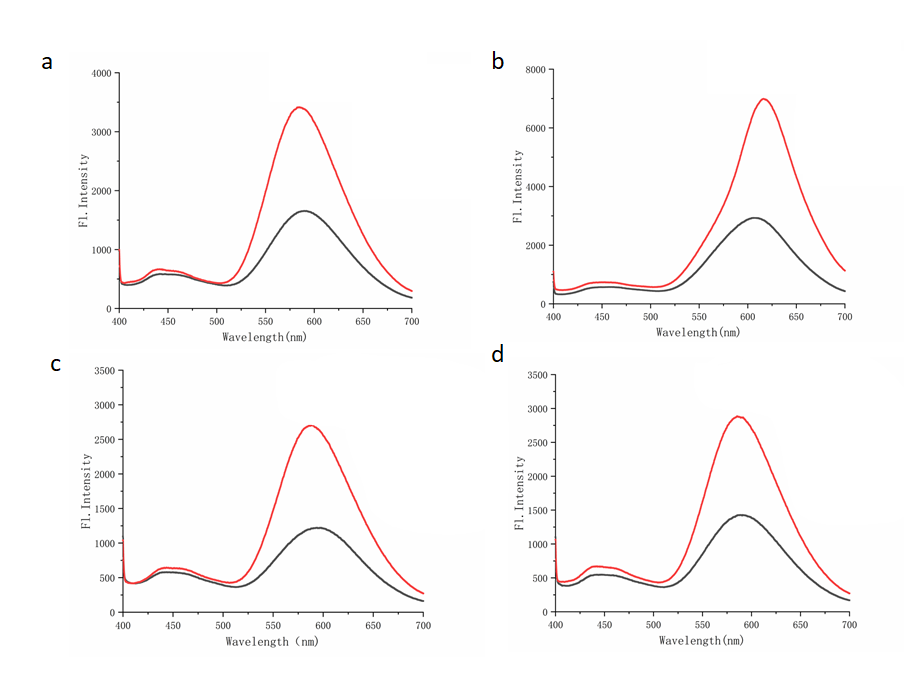


Figure S4. Fluorescence intensity of **HBAE** (10 μM) at 650 nm versus the different cells’ membranes protein. a. U87-MG cell membranes protein, b. N2a cell membranes protein, c.Bend.3 cell membranes protein, d.U251 cell membranes protein. The excitation wavelength was 560 nm.


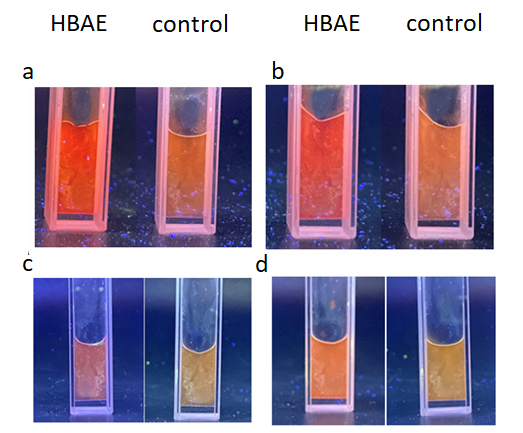


Figure S5.Fluorescence intensity of **HBAE** (10 μM) at 650 nm versus the different cells membranes protein. a. U87-MG cell membranes protein, b. N2a cell membranes protein, c. Bend.3 cell membranes protein, d. HEK293 cell membranes protein.

**Docking scores**

We used the DOCK6.8 in molecular docking^2^, and the docking scores were:

Grid_Score: -42.900635

Grid_vdw_energy: -42.900635

Grid_es_energy: 0.000000

Internal_energy_repulsive: 22.601223

Our experiments are carried out according to the website (https://dock.compbio.ucsf.edu/DOCK_6/index.htm)

**Cell Viability Assay**

The cytotoxicity of HBAE was studied by CCK8 assay. Briefly, 100 μL of HEK293 cell suspension was placed in a 96-well plate. The plates were preincubated in an incubator for 24 h (37 °C, 5% CO_2_). A volume of 10 μL of different concentrations of **HBAE** was added to the plates. The plates were incubated for 24 h in an incubator. A volume of 10 μL of CCK8 solution was added to each well. The plates were incubated for 2 h in an incubator. The absorbance at 450 nm was measured with a microplate reader to reflect the cell viability of each well.


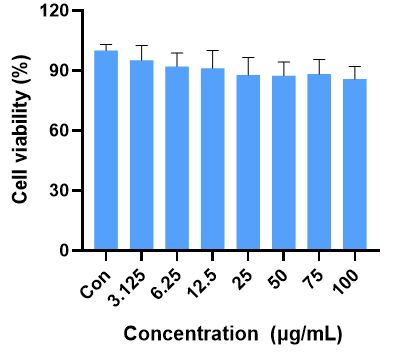


Figure S6. CCK8 of the cell viability of **HBAE**. (Each sample was tested using three replicates, and the results are reported as the mean ± standard deviation).

**Cell line**: U87-MG，Bend.3, N2a, HEK293 cells were maintained in DMEM (Thermo Fisher Scientific, USA), supplemented with 10% FBS (Vivacell, Shanghai, China) and 1% penicillin−streptomycin (Thermo Fisher Scientific, USA). Cells were incubated at 37 °C with 5% CO_2_.

**Western blot analysis**

Western blot analysis was performed on the 4 cell lines to determine the expression of BACE1. β-actin antibody (1:1000) was used as a normalization control.


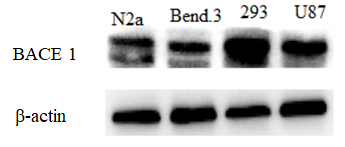


Figure. S7 Western blot images of BACE1 in 4 cell lines.

**Establishment of blood-brain barrier in vitro**

Transwell orifice plate was used in the experiment. 10000 Bend.3 cells were inoculated into each well in the upper chamber. Complete culture medium was added into the lower chamber, and the solution was changed every 48 hours. The resistance was measured by trans endothelial resistance meter (TEER). When the resistance value was greater than 200 Ω / cm^2^, the in vitro blood-brain barrier was successfully established. U87-MG cells were inoculated into the lower chamber, adhered to the wall overnight, and **HBAE** were added into the upper chamber after 1, 2 and 4 hours respectively, The lower ventricular cells were fixed with paraformaldehyde, washed with PBS for three times, and stained with DAPI staining solution. After washing with PBS for three times, the BBB ability of the probe was observed under fluorescence microscope.


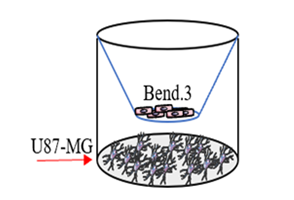


Figure. S8 Establishment of blood-brain barrier in vitro.

**Animal Model**

The animal experiment was approved by the Animal Ethics and welfare Committee, at the Second Xiangya Hospital, Central South University (No.2021595). All animal studies were carried out using the Institutional Animal Care and Use Committee (IACUC) approved procedures. The 22-month-old 5XFAD mice and wild-type mice were ordered from the Jackson Laboratory (34840), and maintained under standard conditions. The animals were housed in sterile cages within laminar airflow hoods in a specific pathogen-free room with a 12 h light/12 h dark schedule and fed autoclaved chow and water ad libitum.

**Real-time in Vivo imaging in 5XFAD Mice of HBAE**

5XFAD mice (n=4, C57 BL/6, 5XFAD, 22-month-old) and an age-matched wild-type mouse (n=4, C57 BL/6, 22-months-old) were shaved before background imaging and were intravenous injected with **HBAE** (2.0 mg kg^-1^, 1% DMSO, 99% PBS, 200 μL). Fluorescence signals from the brain were recorded at different time points after intravenous injection of **HBAE**. For the measurement, a filter set (λex = 560 nm, λem = 680 nm) was used, and optical images were acquired using an exposure time of 1 s. During the imaging process, the mice were kept on the imaging stage under anesthesia with 2.5% isoflurane gas in an oxygen flow (1.5 L/min).


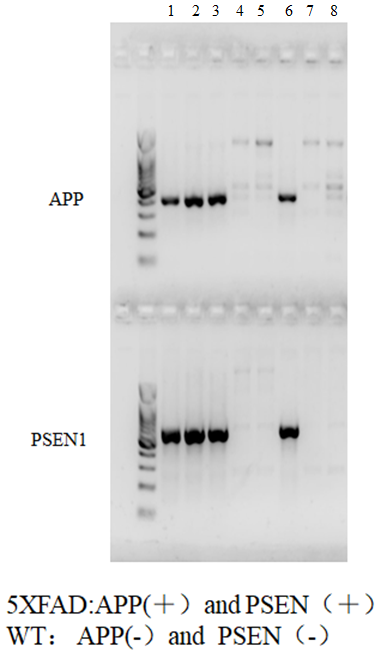


Figure. S9 Gene banding of AD and wild-type mice model. 1, 2, 3 and 6 belong to AD model mice, and 4, 5, 7 and 8 belong to wild-type mice.

**Safety and biocompatibility potentials of HBAE**

In order to study toxicity or biocompatibility results for cellular or in vivo use of developed HBAE probe. The hemolysis test of HBAE was firstly evaluated carefully.

1. Use EDTA anticoagulant tube to take 1mL blood from mouse heart, add 1mL normal saline and mix.

2. Weigh 3 mg of HBAE, add 50 uL DMSO to dissolve a high concentration solution, and prepare 12.5, 25, 50, 100, 200, 400 μg/mL HBAE solution by saline, the positive control (water), and the negative control (normal saline) are placed in a 37 ^o^C water bath for 30 minutes, and 0.2 mL diluted whole blood is added into each tube.

3. Continue to bathe at 37 ^o^C for 60 minutes, take out the Ep tube after the water bath, put it into the centrifuge for centrifugation of 800 g for 5 minutes, observe and take photos.


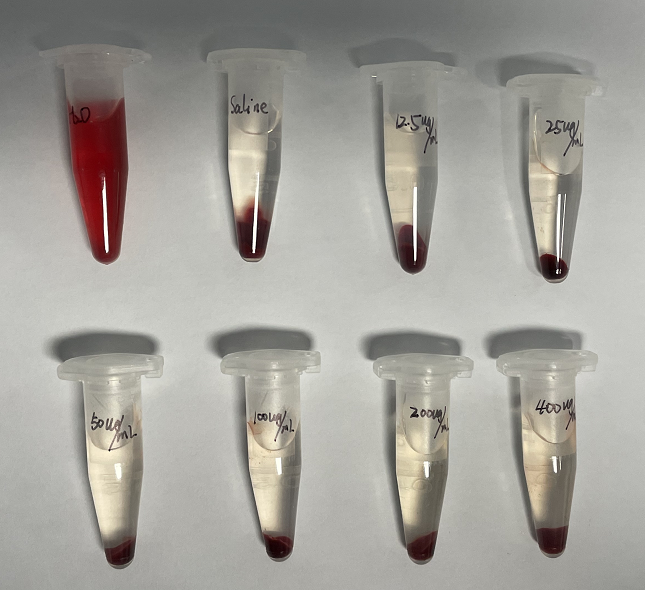


Figure S10. Test of hemolysis activity of formulations. (A) Photos of hemolysis after incubation with different formulations. HBAE of different concentrations are 12.5, 25, 50, 100, 200, 400 μ g/mL, respectively.

b) In vivo safety evaluation:

The ten C57 BL/6 male mice were randomly selected and divided into two groups: normal saline group and HBAE group. C57 BL/6 male mice of HBAE group were intravenous injected with HBAE (2.0 mg kg-1, 1% DMSO, 99% saline, 200 μL). The mice were anesthetized 24 hours after administration, blood was taken from the heart, and serum was collected to investigate liver function indicators ALT, AST and kidney function indicators BUN, Cr. Collect the main organs (heart, liver, spleen, lung, kidney), fix them with 4% paraformaldehyde, embed them in paraffin, and conduct H&E staining on the slices to observe the histopathological changes.

There is not obvious histopathological changes were found in the HE staining observation compared with the normal saline group, indicating good biocompatibility in vivo.


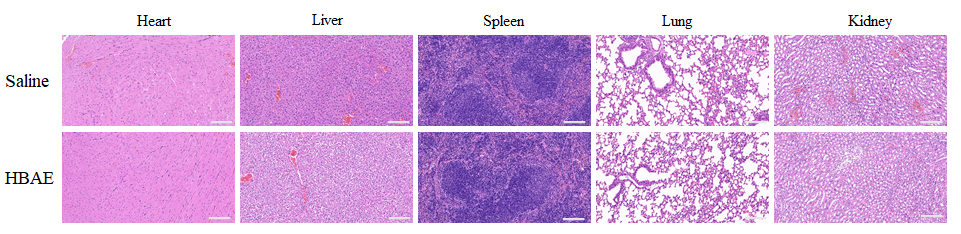
 Figure S11. H&E staining of heart, liver, spleen, lung, kidney tissues of C57 BL/6 mice after HBAE treatment, scale bar = 200 μm.


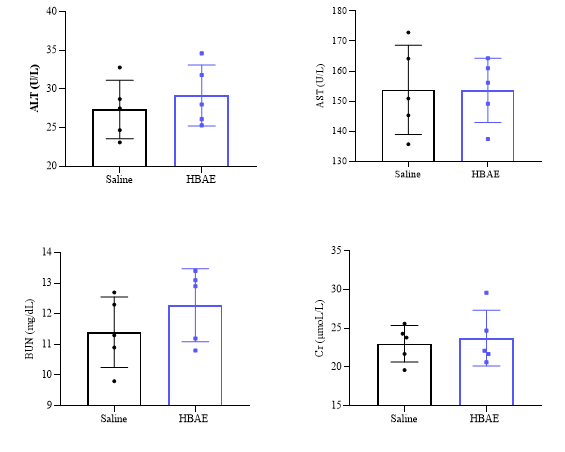


Figure S12. Analysis of serum levels of ALT, AST, BUN and Cr in tumor-bearing mice after HBAE treatment. Data were mean ± SD (n = 5).

The liver function indexes ALT and AST and kidney function indexes BUN and Cr in serum had no significant difference compared with the normal saline group, indicating that HBAE would not affect the liver and kidney functions.

c) The HE staining of the AD brain.

In order to verify that the probe does not damage or damage the brain tissue of mice, the frozen brain sections of AD model mice were stained with H&E staining. It was confirmed that the probe did not damage the brain of AD mice.


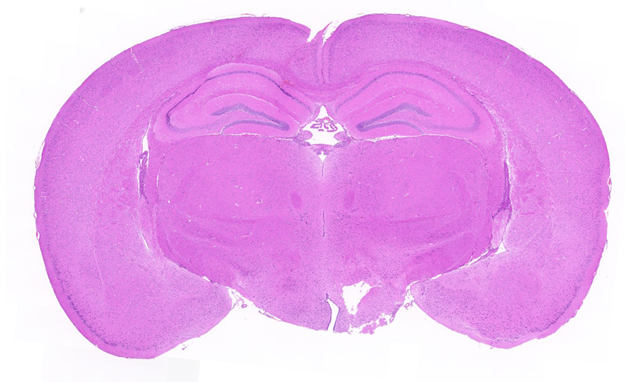


Figure S13. The H&E staining of the AD mice brain after HBAE treatment.


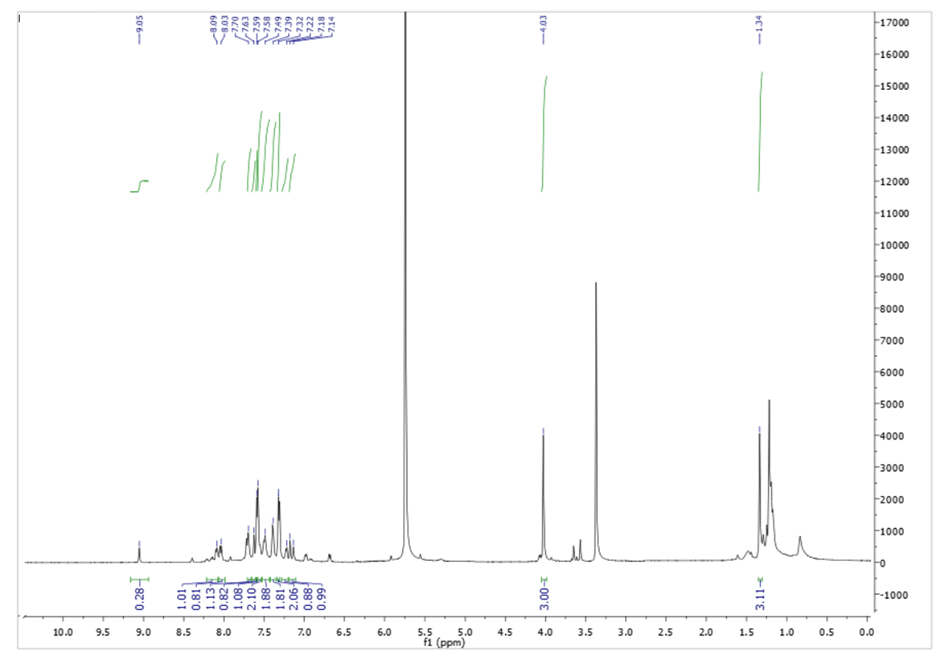


Figure. S14 ^1^H NMR (500 MHz, DMSO-d6) of HBAE.


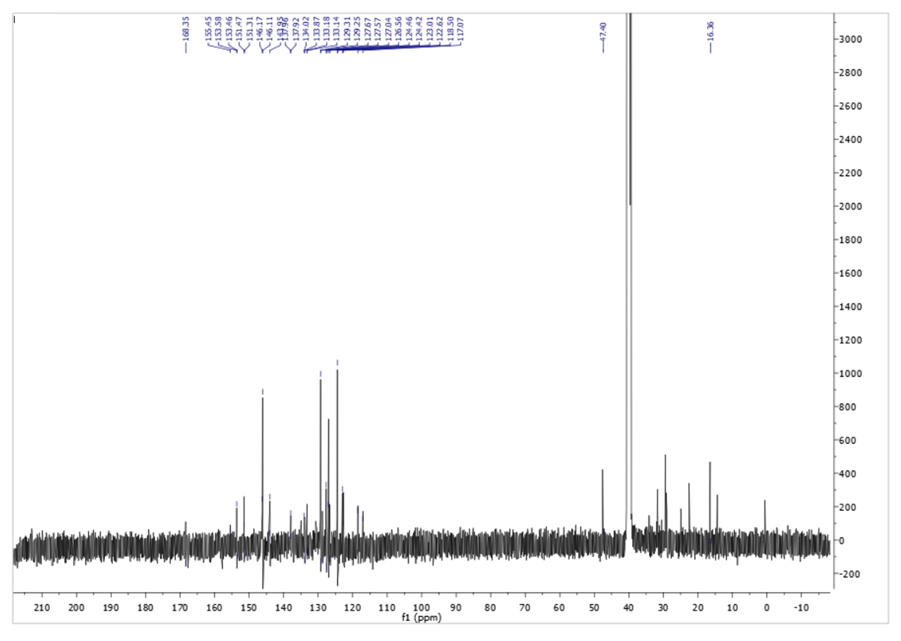


Figure. S15 ^13^C NMR (125 MHz, DMSO-d6) of HBAE.


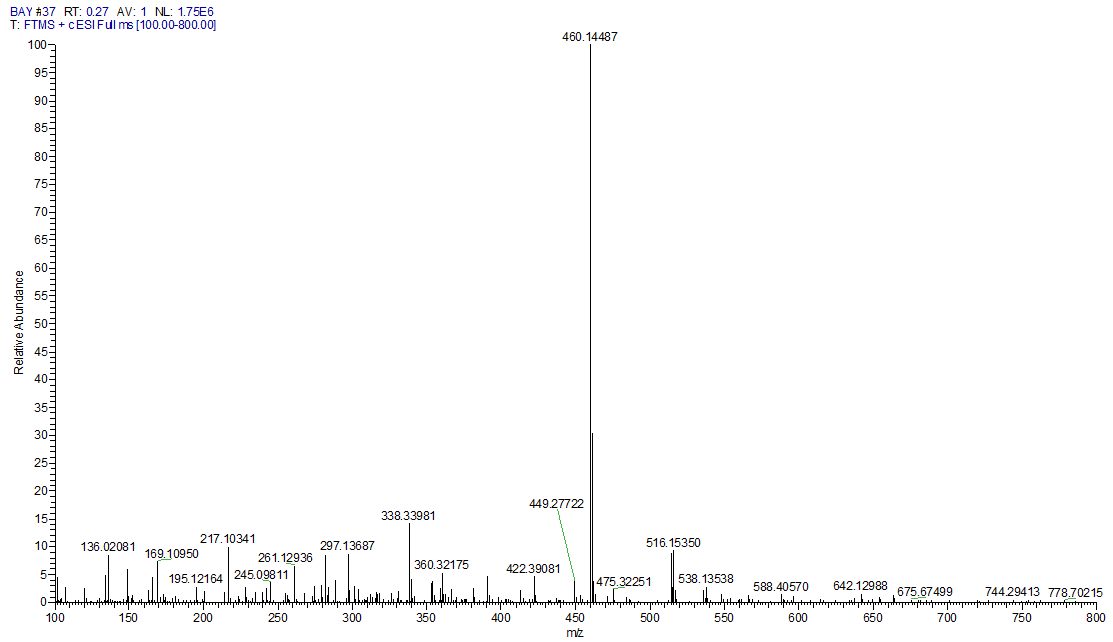


Figure. S16 MS spectra of HBAE.

Reference

1. D. Magde, G. E. Rojas and P. G. Seybold, Photochemistry and Photobiology, 1999, 70, 737-744.

2. Allen WJ, Balius TE, Mukherjee S, Brozell SR, Moustakas DT, Lang PT, Case DA, Kuntz ID, Rizzo RC. J Comput Chem. 2015, 36, 1132-56.
